# The Impact of Urban Culture on Street Vending: A Path Model Analysis of the General Public's Perspective

**DOI:** 10.3389/fpsyg.2021.831014

**Published:** 2022-02-14

**Authors:** Salem A. Al-Jundi, Haitham A. Al-Janabi, Mohammad Asif Salam, Saleh Bajaba, Shakir Ullah

**Affiliations:** ^1^School of Business, Skyline University College, Sharjah, United Arab Emirates; ^2^Faculty of Administrative Sciences, University of Al-Mashreq, Baghdad, Iraq; ^3^Department of Business Administration, Faculty of Economics and Administration, King Abdulaziz University, Jeddah, Saudi Arabia; ^4^Faculty of Finance, University of Maryland Global Campus, Adelphi, MD, United States; ^5^Faculty of Finance, University of Louisiana Monroe, Monroe, LA, United States

**Keywords:** street vending, urban culture, consumption, resistance, microfinance, mediation, PLS-SEM

## Abstract

This study examined the relationship between urban culture and street vending. Prior research on this topic is limited and inconclusive. Therefore, we have proposed an integrated model to test the positive effect of urban culture on street vending using multiple mediations of consumption patterns, resistance, and microfinance. We tested a sample of 425 responses that reflect the public opinion in Baghdad, Iraq. These responses were collected between September and November 2018. A partial least squares–based structural equation modeling is employed to test the validity of measurement models and the significance of the entire structural model, predictive power, and mediation analysis. We found that resistance mediates the effect of urban culture on street vending; low-income consumption and resistance sequentially mediate the effect of urban culture on street vending, while resistance mediates the effect of a lack of microfinance on street vending. The direct impact of culture on street vending is not significant, and a lack of microfinance positively influences the pervasiveness of trading on streets. This study contributes to the extant literature as it proposed and tested a novel and comprehensive model to analyze the relationship between urban culture and street vending, simultaneously examining the effects of culture, consumption, resistance, and microfinance on street vending.

## Introduction

Unlicensed street vendors occupy public spaces and traditional markets, creating problems for residents, pedestrians, formal retailers, and public authorities. They sometimes cause conflicts in society, potentially leading to violence (Tonda and Kepe, 2016). Moreover, they often employ children, working individually or with their parents (Estrada, 2016), and are frequently accused of drug trading and counterfeiting (Ilahiane and Sherry, 2008). On the other hand, in many countries, the informal economy, which consists mainly of street vending, plays a crucial role in income generation, employment creation, and production (Recchi, 2020).

There are no accurate data for street vending or for the informal economy in general due to the fact that street vending and/or informal sector are informal activities operating without registration and licenses. According to the conceptualization of the International Labor Organization (ILO), the formal economy consists of government entities in addition to registered private units with fixed premises, while the informal sector includes unregistered business units such as street vending, agricultural family production, daily construction work, and home-based enterprises (OECD/ILO, 2019). An indicator of the scale of street vending is that informal employment accounts for 42% of total nonagricultural employment in Thailand (2010), 50% in Argentina (2009), 61% in Ecuador (2009), and 70% in Zambia (2008) as estimated by the ILO (ILO, 2013). If we add the small family farms, the informal sector represents a huge part of the entire economy in most developing countries.

The study chose Baghdad, Iraq as a sample to analyze the relationship between culture and street vending for multiple reasons. First, street vending is a crucial part of the vibrancy of cities like Baghdad, Iraq's capital. Second, Iraqis often buy from and trust peddlers; most of the time, the public authorities ignore them. Third, the pervasiveness of street vending has increased dramatically over the last 15 years in the wake of political changes. Since the occupation of Iraq by the US-led alliance in 2003, the state and its major institutions have collapsed. Political and social stability has been severely damaged, and the state has mainly allocated its financial resources to fighting terrorism and resolving sectarian tensions. Moreover, the new regime has shifted to a free-enterprise market that has replaced the state as the major source of employment that it used to be during Saddam Hussein's dictatorial regime. As a result, the unemployment rate has increased, especially among young people, and one-fifth of the population has fallen below the poverty line, even though the country is ranked fifth in the world for oil exports. The number of street vendors has increased sharply, and the public authorities have been unable to formalize their status. Government attempts to evict street vendors or destroy their stalls sometimes trigger protests, such as the major demonstration at the beginning of October 2019 against corruption, unemployment, and poor public services. We have therefore chosen to investigate this widespread and problematic issue.

This study examines the relationship between urban culture and street vending, since the literature on this topic is quite sparse (Tamilarai and Angayarkanni, 2016; Wibisono and Catrayasa, 2018). Scholarly research has focused on street vendors who choose their profession willingly for cultural reasons, and who have a spiritual motivation that gives them satisfaction, enabling them to provide high-quality services in the perception of their clients (Wibisono and Catrayasa, 2018). Understanding of this relationship between culture and street vending needs to be enriched, since research has yielded contradictory statistical results (Voiculescu, 2014; Tamilarai and Angayarkanni, 2016; Alvi and Mendoza, 2017; Wibisono and Catrayasa, 2018). Few studies have taken account of the fact that low-income customers prefer to shop in neighboring streets at low prices and to spend only a short time doing so (Yatmo, 2009; Tamilarai and Angayarkanni, 2016). Therefore, when scholars consider low-income consumption as a dimension of urban culture, their statistical results are inconsistent (Steel, 2012a; Trupp, 2015; Tamilarai and Angayarkanni, 2016).

Some researchers have suggested a direct effect of resistance on the pervasiveness of street vending, noting that street vendors, in order to survive, adopt a strategy of resistance, despite restrictive policies (Hanser, 2016; Boonjubun, 2017). Here, we argue that resistance as a mediator is able to explain the relationship between culture (or low-income consumption) and street vending, given that urban culture (or low-income consumption) may not affect street vending directly. For this reason, we propose a mediation model that can be examined theoretically and empirically. The model posits that urban culture positively impacts street vending through low-income consumption and resistance and the mediating effect of resistance on the relationship between a lack of microfinance and street vending.

This study relies on the cultural approach, which argues that street vendors choose their endeavor for cultural reasons rather than on the basis of rational decisions. They establish relationships with their friends and the community on the basis of solidarity and reciprocity, and they successfully build relationships with customers on the basis of trust. They also enjoy freedom and flexibility that allow them to have control over their lives. For their part, customers support street vendors who offer the goods and the services they need at affordable prices (Williams and Gurtoo, 2012; Williams and Youssef, 2014). In this context, the present study examines whether culture impacts street vending directly or indirectly through consumption and resistance.

The model is tested empirically, using a survey of the general public's attitudes toward street vending and corresponding factors in the context of Baghdad. Researchers have reviewed public policies on street vending on the basis of national data (Ilahiane and Sherry, 2008; Lyons, 2013) and have interviewed street vendors to identify their characteristics (Reid et al., 2010; Tengeh and Lapah, 2013; Wibisono and Catrayasa, 2018). However, there remains a need to understand public opinions on street vending before reviewing public policies on this activity (Chai et al., 2011). The current study formulates a public perspective on this widespread problem in cities, which is a necessary step in developing appropriate legislation.

The study makes significant contributions to the literature on street vending. First, it tests the cultural approach by investigating the direct and indirect effects of culture on street vending. Second, it introduces a distinctive model using sequential mediation analysis. Third, the model is expanded by the addition of the mediating effect of resistance on the relationship between microfinance and street vending. Finally, the results can be used to rank the factors that drive the pervasiveness of street vending in order of importance, with managerial implications for dealing fairly with this problematic issue in the cities of developing countries.

Section Conceptual Framework and Hypothesis Development introduces the conceptual framework and develops the hypotheses on the basis of a thorough review of the literature. Section Methodology sets out the sampling and data collection procedure, derives measurement items for the constructs of the model, and provides a rationale for using a partial least squares structural equation modeling (PLS-SEM) approach to analyze the data. Section Results tests the model and hypotheses and reports the results. Section Discussion and Conclusion discusses the theoretical and managerial implications of the findings, and then considers the limitations of the study and future research directions.

## Conceptual Framework and Hypothesis Development

Scholars often consider the informal economy as an indicator of economic underdevelopment or as an obstacle to economic development. However, in developing and low-income countries, the informal sector increasingly contributes to the elimination of unemployment and poverty (Ilahiane and Sherry, 2008; Lyons, 2013). Street vendors (hawkers or peddlers), as a main element in the informal economy, have existed for decades (Nani, 2016). They are continually at risk of eviction from sidewalks and crowded markets (Recio and Gomez, 2013) because public officials tend not to appreciate the role of hawkers, although their businesses play a major role in the informal economy, contribute to the vibrancy of cities, and form an obvious part of the general economy (Khan and Quaddus, 2020).

**Figure 1:**
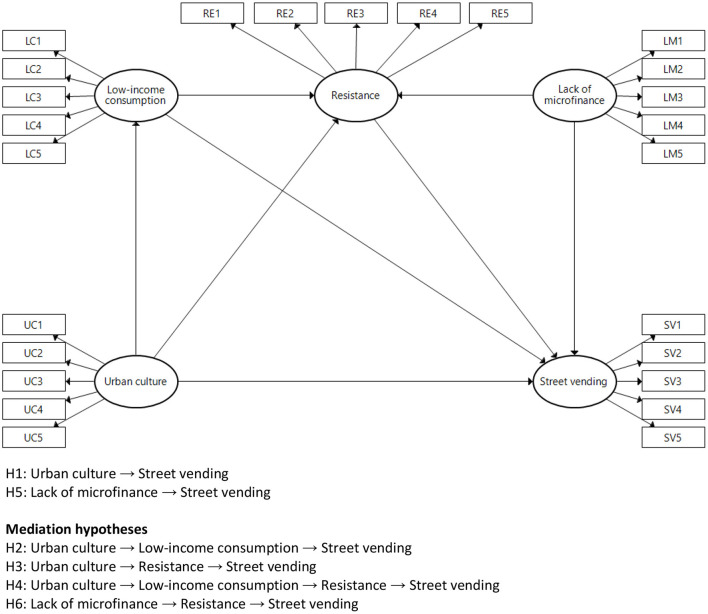
Conceptual research model.

Street vendors earn a low level of income and must compete with formal sellers (Agadjanian, 2002). It is worth noting that there are often too many vegetable sellers competing with each other in overcrowded areas. In this connection, we should differentiate between licensed and unlicensed street vendors. While licensed sellers enjoy a formal relationship with municipal authorities and public officials, unlicensed vendors work under precarious conditions, struggling to avoid eviction from the public streets (Cuvi, 2016). Our study analyzes the impact of urban culture on the pervasiveness of unlicensed street vending via consumption patterns and resistance, in addition to the impact of a lack of microfinance on street vending via resistance. The conceptual research model (Figure 1) and its hypotheses are rooted in the literature, as the following subsections demonstrate.

### Urban Culture

Urban or street culture refers to values and practices shared by the residents of cities. Street vending is a core part of this culture. As we have observed in Baghdad, customers visit nearby traditional markets not only to make purchases but also to spend time communicating with each other, meeting their friends, walking, and looking at the attractive offerings of street vendors. Meanwhile, street vendors are reluctant to move into the formal sector, preferring the hazardous conditions of the informal sector to the relative safety of formal activities (Alvi and Mendoza, 2017). It is this preference that enables them to tolerate the difficulties they encounter (Tamilarai and Angayarkanni, 2016). Some vendors love the flexible spaces and movement; they are voyagers who carry their emotions and dreams with them as they explore new landscapes (Voiculescu, 2014). They enter the informal economy for cultural reasons, such as continuing a traditional family activity, and for social or lifestyle reasons (Williams and Gurtoo, 2012). The urban culture of a city also determines the traditional recipes and eating habits that match the food offered by street vendors (Wardrop, 2006), and the public streets where readers and sellers of newspapers come together represent shared cultural interests (Reuveni, 2002). Thus, we hypothesize:

**H1**. Urban culture positively impacts street vending.

### Low-Income Consumption

Street vendors cannot compete with retail shops in terms of quality, brand name, or variety of products; instead, they attract customers who intend to spend only a short period of time shopping and buy at low prices (Tamilarai and Angayarkanni, 2016). Traditional markets combine both retail shops and street vendors who intentionally locate their business in crowded areas. For instance, low-income customers who cannot afford to go to restaurants buy cooked food from sellers on the streets. Even though their business is somewhat threatened by the formal food retail industry, those sellers continue to provide food to those consumers. Generally, the vendors themselves try to understand changes in customers' needs and to select appropriate public places to reach certain groups of customers, such as tourists (Steel, 2012a). For example, souvenir vendors have become a core part of the tourism economy in countries such as Thailand (Trupp, 2015). Nowadays, vendors increasingly use social media platforms to disseminate information about their business, communicate with nearby customers, and persuade them. Then, customers often respond positively to purchasing from those vendors (Wang et al., 2021b).

Customers enjoy purchasing the products offered in the public streets and traditional markets, since this consumption pattern reflects their values and beliefs. For example, food consumption habits and styles are determined by geographical location, climate, and what foodstuffs can be produced locally, with the result that consumption patterns pass from one generation to the next. Specifically, meat consumption is affected by religion, history, and urban culture (Nam et al., 2010). In short, the culture teaches vendors to produce traditional food or drink that appeals to customers. For their part, the customers, again as part of the culture, enjoy buying such food on the public streets and can identify trusted sellers. Therefore, urban culture establishes a pattern of low-income consumption that creates a real demand for the products offered by street vendors. Thus, we hypothesize:

**H2**. Low-income consumption mediates the effect of urban culture on street vending.

### Resistance

Depending on their social networks, street vendors occupy certain traditional markets or sidewalks (Tengeh and Lapah, 2013); that is, they belong to specific tribes or cities, which gives them a degree of power against residents and authorities. Itinerant vendors, for instance, resist in order to be allowed to remain on the sidewalks and in the markets, taking individual and collective action and sometimes organizing protests (Steel, 2012b). Vendors have neither safety nor security, because they face harassment from the local authorities and often have to pay bribes to sustain themselves on the streets (Saha, 2011). When the authorities demolish their stalls, they find ways to return to their sites with a higher level of resistance (Musoni, 2010). Governmental organizations can reduce the level of resistance by introducing justice practices among peddlers by offering sort of support to them such as building infrastructure in order to formalize their business (Rehman et al., 2021).

Informal workers generally do not group themselves into organizations. Thus, they do not have the collective power to negotiate with governmental organizations, such as the police and municipal authorities, or to collaborate to improve their working conditions (Hummel, 2017). Nevertheless, although city authorities have legal powers, street vendors tend to develop a set of strategies for acquiring formal and informal power (Boonjubun, 2017; Forkuor et al., 2017; Hummel, 2017; Te-Lintelo, 2017). Thus, resistance gives vendors the ability to stay on the sidewalks and in the markets despite the objections of city officials and residents (Zhong and Di, 2017).

People who suffer from poverty and unemployment develop their own subculture to resist oppression (T. A. Martinez, 1997). Street vendors who are poor or unemployed find ways to resist and continue their businesses on the public streets so that they can survive; they do so regardless of the concerns of the official authorities. Researchers have argued that certain groups in a society develop their own oppositional cultures that empower them to resist public trends (Ainsworth-Darnell and Downey, 1998). These vendors believe that they have the right to survive in their neighborhood, and that the authorities do not have the right to evict them unless officials arrange alternative employment for them. Consequently, the culture generates values and beliefs in favor of peddlers staying on the public streets, with the approval of customers, and resistance supports them in doing this. Hence, we propose the following hypothesis:

**H3**. Resistance mediates the effect of urban culture on street vending.

Urban culture creates a consumption pattern, especially for low-income customers. This pattern represents real demand for products offered in public spaces and on sidewalks, and street vendors find their businesses profitable because of the willingness of customers to deal with them. The resulting consumption pattern consolidates the persistence of street vendors working in the informal trading sector. For instance, Khan (2017) found that street vendors are distinguished by cheaper pricing and quicker delivery, and that their customers see street vending as conveniently located, with flexible times and rich customization. Since urban culture generates low-income consumption, the real demand for products offered on public streets establishes resistance among vendors, thereby facilitating the survival of their livelihood and justifying their pervasiveness. Hence, we propose the following hypothesis:

**H4**. Low-income consumption and resistance sequentially mediate the effect of urban culture on street vending.

### Lack of Microfinance

The pervasiveness of street vending can also be explained by a lack of microfinance. Husain et al. (2015) found that personal savings constitute the most considerable source of financing for peddlers. Lyons (2013) found that when peddlers find it difficult to secure formal credit facilities from commercial banks and financial funds, they sell their assets or borrow from cooperative organizations. To finance their economic activities and social security, street vendors sometimes borrow money at exorbitant rates of interest (Saha, 2011; Martinez and Rivera-Acevedo, 2018). Therefore, governments should set up specialized organizations to provide financial support to microbusinesses. Likewise, commercial banks should be encouraged to lend to very small businesses, and the loans should be based on knowledge of the market rather than on technical evaluation of the risks; in this context, an intuitive approach to lending will lead to better results than quantitative methods (Malôa, 2013).

Informal sellers are among the poorest people in society. They cannot afford to rent a retail outlet, expand their business, or shift to the formal sector (Tamilarai and Angayarkanni, 2016). Moreover, they do not meet the minimum requirements to apply for a loan, and banks are reluctant to be involved in microfinance. In short, an acute lack of microfinance results in poor and uneducated people trading on the streets, in contrast to a mature and developed financial system, which would create easier channels for financing microbusinesses and give unemployed people the opportunity to set up small formal businesses (Esubalew and Raghurama, 2020). Since most unemployed and poor people have no access to the financial system to obtain loans, they become resistant. Thus, the strong resistance of street vendors can be explained in part by a lack of microfinance, which leads them to stay on the public streets. Therefore, we hypothesize:

**H5**. A lack of microfinance positively impacts street vending.

**H6**. Resistance mediates the effect of a lack of microfinance on street vending.

## Methodology

### Sampling and Data Collection

The measurement items for the constructs in this study, displayed in Table 1, were translated into Arabic, a language that the majority of Iraqis speak. To check the suitability of the items for the Iraqi cultural context, the questionnaire was discussed with five colleagues at the Middle Technical University, Baghdad, and an initial sample of 25 responses was analyzed. The results confirmed that most of the street vendors are Iraqis and that public officials mostly ignore them, although the authorities sometimes evict them from public streets and traditional markets. The results also indicated that most of the street vendors are uneducated, but that some have secondary school certificates, a diploma, or even a bachelor's degree, because unemployment has spread among young people and graduates. We modified the questionnaire in light of these findings, and the results are shown in Table 1 in an English version.

**Table 1:** Measurement model assessment.

**Item wording**	**Code**	**SFL**	**SE**	***t*-value^a,b^**	**α**	**ρ_C_**	**AVE^c^**
**Urban culture (Reuveni**, **2002****; Wardrop**, **2006****; Tamilarai and Angayarkanni**, **2016****; Alvi and Mendoza**, **2017****; Wibisono and Catrayasa**, **2018****)**							
My city is vibrant despite the spread of street vendors.	UC1	0.691	0.036	19.243	0.718	0.814	0.500
Some street vendors offer traditional and delicious food.	UC2	0.710	0.037	19.326			
Some street vendors prefer working on the streets rather than in the formal sector.	UC3	0.502	0.059	8.485			
People enjoy walking and communicating in the traditional markets.	UC4	0.764	0.025	30.392			
People can find interesting books in a special traditional market.	UC5	0.734	0.035	20.711			
**Street vending (Agadjanian**, **2002****; Ilahiane and Sherry**, **2008****; Recio and Gomez**, **2013****; Palacios**, **2016****)**							
Street vendors spread through the streets and markets of the city.	SV1	0.515	0.062	8.347	0.737	0.824	0.500
City authorities do not provide any support to street vendors.	SV2	0.649	0.046	14.108			
Street vendors seek to improve their income without paying attention to the traffic in the street and the movement of pedestrians on the pavements.	SV3	0.800	0.028	28.395			
Street vendors compete with each other in the overcrowded streets and markets.	SV4	0.762	0.039	19.774			
Street vendors are unlicensed by the municipality or other governmental offices.	SV5	0.735	0.037	19.933			
**Low-income consumption (Reid et al.**, **2010****; Steel**, **2012a****; Trupp**, **2015****; Tamilarai and Angayarkanni**, **2016****)**							
^*^Street vendors are so close to my home.	LC1	0.326	0.064	5.083	0.771	0.855	0.597
Goods, such as vegetables and fruits, are somewhat cheaper on the streets than in shops.	LC2	0.747	0.035	21.155			
Street vendors offer delicious and cheap cooked food.	LC3	0.790	0.025	31.818			
I can find souvenirs and accessories at low prices on the streets.	LC4	0.845	0.018	46.089			
Street vendors offer similar goods to shops.	LC5	0.664	0.041	16.208			
**Resistance (Musoni**, **2010****; Steel**, **2012b****; Tengeh and Lapah**, **2013****; Hanser**, **2016****; Boonjubun**, **2017****)**							
Street vendors resist being evicted from sidewalks and traditional markets.	RE1	0.750	0.037	20.009	0.840	0.886	0.610
Street vendors have developed strategies to enable them to stay on the streets.	RE2	0.821	0.024	34.518			
Street vendors will return to their sites if the city's officials demolish their stalls.	RE3	0.786	0.033	23.864			
Street vendors sometimes protest against eviction from the streets.	RE4	0.746	0.038	19.630			
Street vendors occupy certain markets or sidewalks depending on their social networks.	RE5	0.799	0.026	30.762			
**Lack of microfinance (Saha**, **2011****; Lyons**, **2013****; Husain et al.**, **2015****; Tamilarai and Angayarkanni**, **2016****)**							
Street vendors could not get formal credit facilities from commercial banks.	LM1	0.775	0.025	31.259	0.771	0.843	0.518
Street vendors depend on their savings and selling family assets to set up microbusinesses.	LM2	0.740	0.039	19.186			
If street vendors managed to get a loan, they would pay an exorbitant interest rate.	LM3	0.753	0.032	23.643			
Street vendors could not afford the rent of retail outlets.	LM4	0.685	0.041	16.652			
There is no specialized public organization to financially support microbusinesses.	LM5	0.637	0.045	14.164			

*SFL, Standardized factor loadings; SE, Standard error*;

* *Problematic item having SFL <0.500 and removed from final analysis*;

a *Test-statistics are obtained by 5,000 Bootstrap runs*;

b *Absolute t-values > 1.96 are two-tailed significant at 5 percent; α, Cronbach's Alpha; ρ_C_, Composite reliability; AVE, Average variance extracted*;

c *Percentage of variance of item explained by the latent variable*.

Some researchers have interviewed street vendors in order to understand their characteristics and the factors that affect their livelihoods (Reid et al., 2010; Tengeh and Lapah, 2013; Wibisono and Catrayasa, 2018). The current study instead adopts the approach recommended by Chai et al. (2011), with the aim of tracking the attitudes of the general public on the problematic issue of street vending. Their approach is appropriate because the problem affects the social and economic daily life of cities in two opposing ways. On the one hand, it has negative impacts in terms of traffic, competition with the modern retail industry, and violence. On the other hand, it reduces poverty and unemployment. Obtaining a clear understanding of public opinion on the issue is, therefore, a necessary step in reviewing public policies on how to deal fairly with street vendors.

Google Forms were used to administer the electronic survey, which was distributed via a hyperlink sent to participants by e-mail, WhatsApp, or Facebook. We began by inviting students, administrative staff, and faculty members at Middle Technical University, Baghdad to take part. Then, we encouraged our students to ask their friends and relatives outside the university to participate, and we also involved digital friends contacted via social networks. Our aim was to include 600 participants from a range of social classes. In the end, because of limitations of time and resources, we collected 463 responses. We excluded 38 of these on the grounds that the respondents had given the same answer to all the questions. The final sample, therefore, consisted of 425 complete and usable responses collected between September and November 2018. The raw data were deposited at Mendeley and can be viewed at Al-Jundi (2019).

The study adopted a sampling method introduced by Krejcie and Morgan (1970) in order to determine the minimum size of the sample required for a given population. A total number of 384 participants will be required to gain a 95% confidence interval for a population that exceeds one million persons with a marginal error of ±5%. We managed to collect 425 reliable responses that are acceptable, taking into consideration the limitations of this paper (see section Limitations and Recommendations for Further Research). The study, therefore, uses nonprobabilistic sampling with an unlimited population.

Of the participants, 67% were men and 33% were women. In terms of education, 25% had not completed secondary schooling, 41% (most of whom were university students) had a secondary school certificate, 20% had a diploma or a bachelor's degree, and 14% (mainly faculty members) were postgraduates. With regard to monthly household income, 41% earned less than $400, 37% earned $400–999, 12% earned $1,000–1,499, and 10% earned more than $1,500. Participants under the age of 25 accounted for 35% of the sample, while 44% were aged 25–40, and 21% were 41 or older. Thus, the participants come from different educational backgrounds and social classes, which make our sample fairly representative of the general public in the capital city of Baghdad.

### Measurement Variables

In order to test the conceptual research model using PLS-SEM, we constructed measurable (observed) variables that reflect constructs drawn from the literature. All the indicator variables were measured using a seven-point Likert-type scale, shown in Table 1 (1 strongly disagree, 2 disagree, 3 somewhat disagree, 4 neither agree nor disagree, 5 somewhat agree, 6 agree, and 7 strongly agree).

The review of the literature served to identify five items that reflect each construct. The measurement items for the pervasiveness of street vending were drawn from work by Agadjanian (2002), Ilahiane and Sherry (2008), Recio and Gomez (2013), and Palacios (2016), while the observed variables for urban culture were derived from the work of Reuveni (2002), Wardrop (2006), Tamilarai and Angayarkanni (2016), Alvi and Mendoza (2017), and Wibisono and Catrayasa (2018). The items for consumption patterns were derived from Reid et al. (2010), Steel (2012a), Trupp (2015), and Tamilarai and Angayarkanni (2016), and resistance was tracked using indicators proposed by Musoni (2010), Steel (2012b), Tengeh and Lapah (2013), Hanser (2016), and Boonjubun (2017). Finally, the lack of microfinance was measured using indicators introduced by Husain et al. (2015), Lyons (2013), Saha (2011), and Tamilarai and Angayarkanni (2016).

### Statistical Procedures

To validate our proposed model, we adopted a structural equation modeling (SEM) approach for a number of reasons. First, SEM is well recognized among researchers, as many of the concepts of social science are latent variables that can only be measured via observed indicators (Hair et al., 2017, 2019; Latan and Noonan, 2017). Second, SEM is more powerful than factor analysis, path analysis, or multiple linear regression and has already been used in similar studies (Al-Jundi et al., 2019, 2020; Shujahat et al., 2020; Ali, 2021; Ali et al., 2021a,b; Wang et al., 2021a). Third, SEM takes into consideration measurement error in the observed variables involved in a corresponding model (Fornell and Larcker, 1981). Fourth, PLS-SEM allows the examination of causal relationships among many latent variables simultaneously, as well as the calculation of direct and indirect effects of a complex model. Finally, SEM gives a complete picture of the entire model, regardless of the complexity of the relationships among the constructs and observed variables.

There are two approaches to estimating such a model: a covariance-based SEM (CB-SEM) approach and a partial least squares SEM (PLS-SEM) approach. CB-SEM presumes a multivariate normal distribution and seeks to identify the model parameters that minimize the discrepancy between the estimated and sample covariance matrices. PLS-SEM attempts to maximize the explained variance of the endogenous constructs (Hair et al., 2017). The current paper uses the PLS-SEM technique for four reasons. First, PLS-SEM estimates a complex model with many constructs, observed variables, and path model relationships to guarantee convergence regardless of sample size and distribution assumptions (Gefen and Straub, 2005). Second, PLS-SEM focuses on prediction, which allows the derivation of managerial implications. Third, PLS-SEM is suitable for developing a theory (Hair et al., 2017, 2019). Finally, PLS-SEM is recommended for the estimation of mediation models, including sequential mediation analysis (Sarstedt et al., 2020).

## Results

### Assessment of the Measurement Model

In the initial step of the factor analysis, item loadings above 0.700 were retained and those below 0.40 were deleted (as recommended by Hair et al., 2017). All standardized factor loadings were higher than the cut-off value of 0.707. We found that the item loading for LC1 was below 0.40, and we therefore deleted it. Table 1 shows the outer loadings of all the constructs in the study.

To examine internal consistency reliability, we used Cronbach's alpha (α) and composite reliability (ρ_C_) for all the constructs. The rule of thumb indicates that α and ρ_C_ should be above 0.700. As Table 1 shows, these requirements were met for all the constructs.

To assess convergent validity (construct communality), we used average variance extracted (AVE), which is calculated as the mean value of the squared outer loadings associated with each construct (Gefen and Straub, 2005; Hair et al., 2017). As Table 1 shows, the AVE for all constructs exceeds the critical cut-off point of 0.500 (Latan and Noonan, 2017), thus ensuring convergent validity.

To establish discriminant validity, the heterotrait–monotrait ratio (HTMT) was used. If the value of HTMT is lower than the threshold value of HTMT_0.85_ (the conservative cut-off point) or HTMT_0.90_ (the liberal cut-off point), discriminant validity is established (Henseler et al., 2014). Table 2 shows that the HTMT ratios among the constructs are all below the cut-off point of HTMT_0.85_, and discriminant validity is thus established.

**Table 2:** Assessment of discriminant validity using HTMT.

**Constructs**	**1**	**2**	**3**	**4**	**5**
1.Urban culture	(0.900)	0.185^**^	0.567^**^	0.491^**^	0.374^**^
2. Street vending	0.246 [0.181, 0.387]	(0.900)	0.133^*^	0.479^**^	0.376^**^
3. Low-income consumption	0.735 [0.639, 0.823]	0.181 [0.137, 0.319]	(0.900)	0.404^**^	0.292^**^
4. Resistance	0.607 [0.497, 0.704]	0.592 [0.475, 0.695]	0.508 [0.382, 0.620]	(0.900)	0.485^**^
5. Lack of microfinance	0.465 [0.364, 0.577]	0.454 [0.328, 0.583]	0.383 [0.260, 0.499]	0.576 [0.463, 0.668]	(0.900)
Mean	4.977	5.584	5.020	5.655	5.359
SD	1.201	1.323	1.258	1.121	1.208

*Brackets show the lower and upper bounds of the 95% BCa confidence intervals; The diagonal lines indicate the cut-off values for HTMT; Above the diagonal elements are the correlations between the construct; Correlation significance levels*:

* *p < 0.05*;

** *p < 0.01*.

### Predictive Relevance of the Model

To analyze the model's predictive relevance, we distinguished between in-sample prediction (explanatory power) and out-of-sample prediction (predictive power). Explanatory power can be evaluated using the coefficient of determination (*R*^2^), which indicates the predictive accuracy. As a rule of thumb, *R*^2^ values below 0.25 are considered weak. Table 3 shows that the *R*^2^ values for street vending (0.267), low-income consumption (0.321), and resistance (0.361) can be considered moderate; that is, more than 25% of the amount of variance in all the endogenous constructs is explained by the corresponding exogenous constructs. These results are acceptable in the context of research in the behavioral and social sciences (Hair et al., 2017).

**Table 3:** Determination coefficients (*R*^2^) and predictive relevance (Q^2^) of endogenous (omission distance = 7).

**Endogenous variable**	***R^**2**^* values**	**Threshold**	***Q^**2**^* values**	**Threshold**
Street vending	0.267	≥0.25 (weak)	0.121	>0
Low-income consumption	0.321	≥0.50 (moderate)	0.185	
Resistance	0.361	≥0.75 (substantial)	0.213	

The effect size *f*^2^ assesses how strongly an exogenous variable participates in explaining a target endogenous variable in terms of *R*^2^. As a rule of thumb, *f*^2^ values of 0.02, 0.15, and 0.35 are weak, moderate, and large, respectively (Hair et al., 2017). **Table 5** shows that urban culture has a strong effect size in explaining low-income consumption. Lack of microfinance has a moderate effect on resistance, which is similar to the effect of resistance on street vending. Microfinance, consumption, and culture have weak effects on their target constructs, whereas urban culture and consumption pattern have no effect on street vending.

Even though the data collected reflect the general public's perspective from the capital city of Iraq, the quality of predictive power of the proposed model helps to generalize conclusions and drive managerial implications. To test the predictive relevance of the endogenous variables, we used a blindfolding procedure. Table 3 gives the Q2 values for our endogenous latent constructs. Applying the same rule of thumb used for effect size, we find that street vending has weak predictive power and that the power of low-income consumption and resistance is moderate. All the endogenous variables have Q2 values greater than 0, which provides evidence of the model's predictive relevance (Geisser, 1974; Stone, 1974; Hair et al., 2019). Accordingly, we can safely generalize the conclusions derived from this study, taking into consideration the limitations raised in section 5.4.

### Structural Model Assessment

As an initial step, we used the variance inflation factor (VIF) as an indicator of collinearity in the structural model. Table 4 shows that all the VIF values are below the cut-off value of 3.00. Thus, there are no collinearity issues in the structural model.

**Table 4:** Variance inflation factors (VIF) as an indicator of collinearity.

**Endogenous variables**	**Low-income consumption**	**Resistance**	**Street vending**
Lack of microfinance		1.183	1.365
Low-income consumption		1.525	1.564
Resistance			1.534
Urban culture	1	1.637	1.731

**Figure 2:**
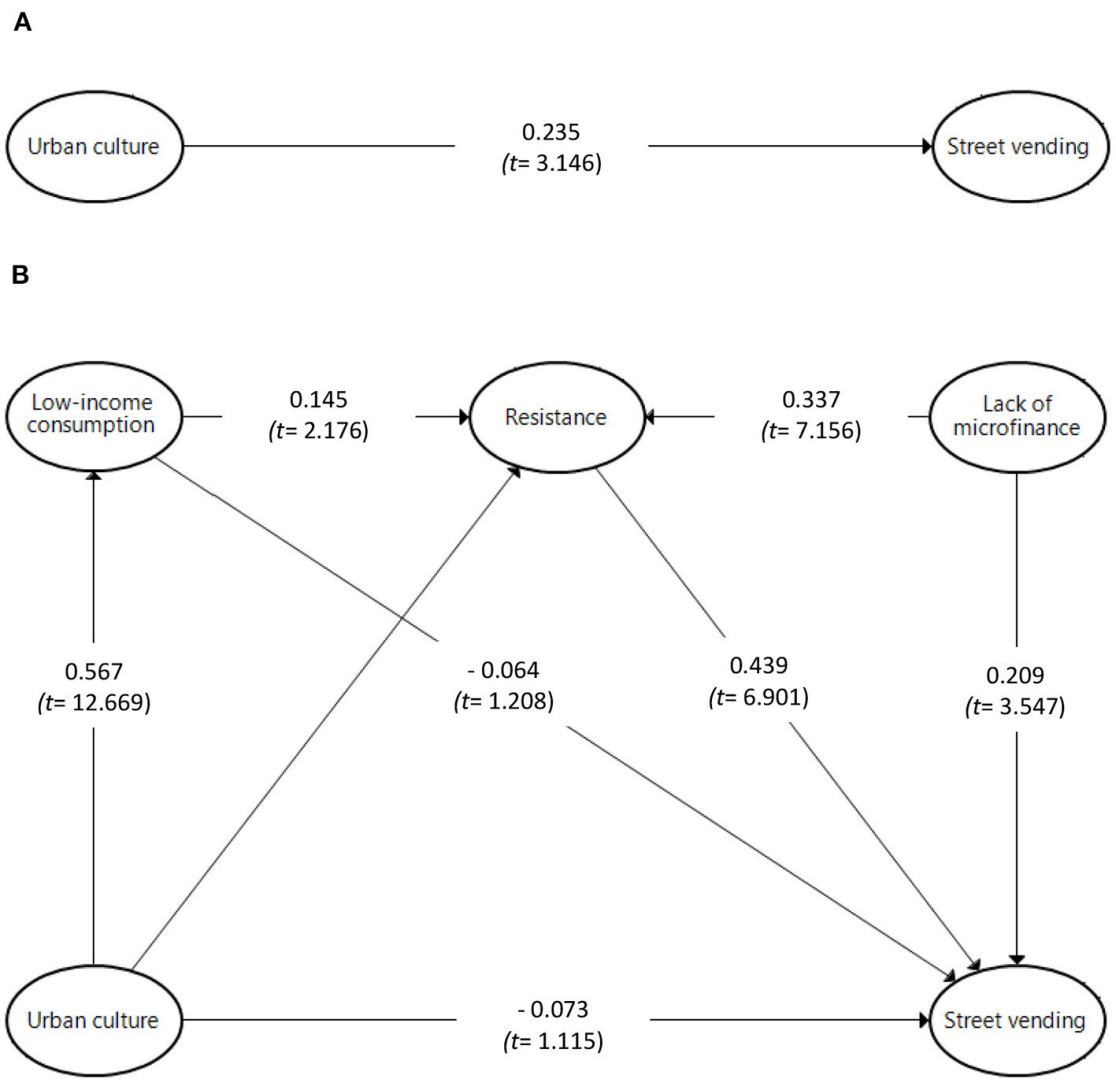
Structural model results. **(A)** Model with total effect. **(B)** Model with double mediations.

To test the significance of the path coefficients, we ran bootstrapping of 5,000 iterations (subsamples) at 95% bias-corrected confidence intervals. The empirical results for all the direct paths in Table 5, Figure 2B, are significant, with the exception of the direct effect of urban culture on street vending and low-income consumption on street vending. The former finding suggests that H1 is not supported. The empirical results also show that a lack of microfinance has a positive and significant effect on street vending, which provides support for H5.

**Table 5:** Construct effects on endogenous variables.

**Structural path**	**Path coefficient**	**Significant difference (*p* < 0.05)?**	**95% BCa confidence interval**	***f* ^2^**	**Conclusion**
Urban culture → Street vending	−0.073^ns^	No	[−0.173, 0.040]	0.004	
Urban culture → Low-income consumption	0.567^***^	Yes	[0.482, 0.634]	0.473	
Urban culture → Resistance	0.283^***^	Yes	[0.172, 0.386]	0.079	
Low-income consumption → Street vending	−0.064^ns^	No	[−0.151, 0.027]	0.004	
Low-income consumption → Resistance	0.145^*^	Yes	[0.039, 0.258]	0.022	
Resistance → Street vending	0.439^***^	Yes	[0.330, 0.539]	0.168	
Lack of microfinance → Resistance	0.337^***^	Yes	[0.256, 0.410]	0.151	
H5 = Lack of microfinance → Street vending	0.209^***^	Yes	[0.110, 0.306]	0.044	Accepted
Assessment of goodness-of-fit model					
Standardized root mean square residual (SRMR) composite model = 0.079					

*ns, Not significant; t (0.05, 4999) = 1.645; t (0.01, 4999) = 2.327; t (0.001, 4999) = 3.092*;

* *p < 0.05*;

*** *p < 0.001; one-tailed test*.

*BCa, Bias-corrected confidence interval. Bootstrapping based on n = 5,000 subsamples*.

### Mediation Analysis

This study followed the updated procedure in Nitzl et al. (2016) to test the mediation hypotheses. Again, the bootstrapping of 5,000 iterations at 95% bias-corrected confidence intervals allowed the indirect effects to be tested. As Figure 2A, Table 6(A) show, urban culture has a significant total effect on street vending (β = 0.235; *t* = 3.146). However, when low-income consumption and resistance are introduced as mediators, urban culture no longer has a significant direct effect on street vending (β = −0.073, *t* = 1.115), as shown in Figure 2B, Table 6(B). This suggests that H1 is not supported.

**Table 6:** Summary of mediating analyses.

**Structural path**		**Path coefficient**	***t-*value**	**Significant difference (*p* < 0.05)?**
**(A) Total effect model**				
Urban culture → Street vending		0.235^***^	3.146	Yes
**(B) Direct effect model (H1)**				
H1 = Urban culture → Street vending		−0.073^ns^	1.115	No
**Indirect effects of poverty on street vending**	**Path coefficient**	**Significant difference (*****p*** **<** **0.05)?**	**95% BCa confidence interval**	**Conclusion**
**(C) Indirect effect model (Single mediation model) (H2 and H3)**				
H2 = Urban culture → Low-income consumption → Street vending	−0.036^ns^	No	[−0.088, 0.015]	Not accepted
H3 = Urban culture → Resistance → Street vending	0.124^***^	Yes	[0.072, 0.188]	Accepted
H6 = Lack of microfinance → Resistance → Street vending	0.148^***^	Yes	[0.100, 0.201]	Accepted
**(D) Indirect effect model (Double mediation model) (H4)**				
H4 = Urban culture → Low-income consumption → Resistance → Street vending	0.036^*^	Yes	[0.009, 0.070]	Accepted
**Assessment of goodness-of-fit model**				
Standardized root mean square residual (SRMR) composite model = 0.079				

*ns, Not significant; t (0.05, 4999) = 1.645; t (0.01, 4999) = 2.327; t (0.001, 4999) = 3.092*;

* *p < 0.05*;

*** *p < 0.001; one-tailed test*.

*BCa, Bias corrected confidence interval. Bootstrapping based on n = 5,000 subsamples*.

The indirect effect of urban culture on street vending via low-income consumption is also not significant (β = −0.036, *t* = 1.183) as shown in Table 6(C), and this indicates that H2 is not supported.

As Table 6(C) shows, the indirect effect of urban culture on street vending via resistance is significant (β = 0.124, *t* = 3.686). This indicates that resistance fully mediates the relationship between urban culture and street vending, and H3 is therefore supported.

The empirical results in Table 6(D) suggest that urban culture is positively associated with low-income consumption, that low-income consumption is positively associated with resistance, and that resistance is related to higher levels of street vending. These results suggest that low-income consumption and resistance are two sequential mediators that fully and jointly mediate the influence of urban culture on street vending. Therefore, H4 is supported.

Table 6(C) shows that the indirect effect of lack of microfinance on street vending via resistance is significant (β = 0.148, *t* = 4.816). This result suggests that resistance partially mediates the relationship between lack of microfinance and street vending, and H6 is therefore supported.

## Discussion and Conclusion

### Discussion of the Results

Surprisingly, the results of this study indicate that urban culture does not have a significant direct effect on street vending (H1). Moreover, the indirect effect of urban culture on street vending via low-income consumption (H2) is not significant. Thus, it seems that culture does not impact street vending. These results contradict previous research (Voiculescu, 2014; Tamilarai and Angayarkanni, 2016; Alvi and Mendoza, 2017; Wibisono and Catrayasa, 2018) and the cultural approach (Williams and Gurtoo, 2012; Ladan and Williams, 2019). Furthermore, the low-income consumption pattern, which can be considered another dimension of culture, does not impact street vending (Table 5), which again contradicts previous research (Steel, 2012a; Trupp, 2015; Tamilarai and Angayarkanni, 2016).

Nevertheless, we find that urban culture has a significant and positive impact on street vending via resistance (H3). This is a case of full mediation, since there is an indirect effect only. Furthermore, urban culture impacts street vending via serial mediation of low-income consumption and resistance (H4), with no direct effect of urban culture on street vending. In short, urban culture has a significant and positive impact on street vending through sequential mediators and is fully mediated.

We also confirm the direct effect of microfinance on street vending (H5) and the indirect effect through resistance (H6). This is a case of complementary partial mediation, since the direct and indirect effects are both positive and significant. Researchers agree that a lack of microfinance has an impact on the pervasiveness of street vending (Saha, 2011; Lyons, 2013; Husain et al., 2015). Even though resistance has a direct effect on street vending (Table 5), as previously established (Musoni, 2010; Tengeh and Lapah, 2013; Hanser, 2016; Boonjubun, 2017), the mediating effect of resistance is more important than the direct effect in explaining the pervasiveness of street vending.

Lastly, the results shown in Tables 5, 6 help to rank the paths in order of importance. First comes the direct effect of resistance on street vending, followed by the total effects of urban culture on street vending, the direct effect of a lack of microfinance on street vending, the indirect effect of microfinance on street vending through resistance, and the indirect effect of urban culture on street vending through resistance, in that order.

### Theoretical Implications

This study contributes to the literature in several ways. First, it proposes and tests a new and comprehensive model to analyze the relationship between urban culture and street vending, simultaneously examining the effects of culture, consumption, resistance, and microfinance on street vending. Second, it investigates the general public's perceptions of the issue of street vending as a problem facing cities in developing countries, which is a necessary step in reviewing public policies and determining how to deal fairly with street vendors. Third, it develops measurement variables for the constructs in question, some of which are used for the first time, and confirms that they are reliable and valid. Fourth, the statistical analysis contradicts the findings of previous studies and sheds light on the cultural approach by showing that urban culture and low-income consumption (as another dimension of urban culture) have no significant direct effect on street vending. Finally, the study offers three novel and important findings: (1) Urban culture positively influences street vending via resistance; (2) Urban culture impacts street vending via serial mediation of low-income consumption and resistance; (3) Microfinance positively impacts street vending directly or through resistance. These findings are the main contributions of this study, and they will enrich the cultural approach. In short, urban culture (in the form of consumption patterns) impacts the pervasiveness of street vending if we take into consideration the mediator of resistance.

### Managerial Implications

Because the predictive relevance of the model has been established, we can safely derive the following managerial implications. The results described in the previous section are of direct relevance to both public entities and scholarly researchers, as they allow the driving factors of the pervasiveness of street vending to be ranked in order of importance: first, resistance; second, urban culture; and third, lack of microfinance (see Tables 5, 6). Resistance is formed by three important observed indicators in sequence, as the standardized factor loadings in Table 1 indicate. First, street vendors develop strategies to enable them to stay on the streets; second, they depend on their social networks; and third, they return to their sites following the demolition of their stalls. Public policy must therefore recognize that the eviction of street vendors from public spaces is not a solution (Batréau and Bonnet, 2016), and policymakers should seek other ways of formalizing street vending.

There are two main factors that shape urban culture: people enjoy walking and communicating in the traditional markets, and they can find interesting items, such as books, in specialized markets. These cultural factors give sellers two sets of incentives to continue trading informally on sidewalks. First, the societal culture, represented by urban culture and patterns of consumption, creates a real demand for the goods and services offered in public spaces, and this encourages sellers to continue trading on the public streets. Second, because they cannot find jobs in the formal sector, street vendors have only one way to earn income, namely by working hard on the streets (Onodugo et al., 2016). In other words, street vendors fulfill their own and their customers' needs, and the culture cannot be changed in the short term.

In terms of lack of microfinance, the most significant factor is that vendors have no access to formal credit facilities. To address this problem, municipal authorities should build infrastructure that is specifically designed to formalize street vendors; for example, they can construct special areas for vendors (Te-Lintelo, 2017) and legalize trading between the informal and formal sectors. The banking sector should be encouraged to adopt a new approach to risk that would enable them to offer loans to microbusinesses (Malôa, 2013), and the public authorities should provide financial support so that poor and unemployed people can set up formal microbusinesses.

We have learned from this research that street vendors are part of the vibrancy of many cities in developing countries. They play an important role in society by providing a range of products to low-income customers. They also help to eliminate poverty and unemployment, enabling people to depend on their own resources when governments fail to tackle those problems (Onodugo et al., 2016). Furthermore, Street vendors cause many problems to traffic flows and suffer from the harmful environment when doing their business such as noise and air pollution. The phenomenon cannot be avoided even the government would evict them from streets and public spaces. The unemployment and poverty immediately pushed them to return. The problem is pervasiveness because at least it has roots in urban culture, consumption patterns, resistance, and lack of microfinance. The best solution to this problematic issue is that the government should invest to formalize the informality of street vending. Therefore, we can increase their contribution to the economic advances and decrease their negative impact on cities.

### Limitations and Recommendations for Further Research

Although the path coefficients of the relationship between constructs and the predictive relevance of the entire model are statistically significant, the results of this study are subject to a number of limitations. First, the study uses non-probabilistic sampling with an unlimited population, and the sample of 425 responses can be considered small in the context of the total population of Baghdad. The results would be more accurate if we could increase the sample so that it is more representative of the population as a whole. Second, because we collected the raw data via the Internet and social media, we cannot guarantee the full engagement of the participants. Third, the study relates specifically to the context of Iraq, a country that has suffered recent political instability. Thus, it is important to apply the model to data drawn from other cities and countries with different political circumstances.

Fourth, the model is limited to examination of the impact of culture on street vending. Future research should examine the multivariate impact of other important antecedents of street vending, such as poverty (Estrada and Hondagneu-Sotelo, 2011; Saha, 2011), unemployment (Truong, 2018), education (Williams and Gurtoo, 2012; Husain et al., 2015), and immigration (Lapah and Tengeh, 2013). The inclusion of the moderating effects of gender, income, and educational background would improve the model conceptually and statistically. Scholars should also revisit the cultural approach and other theories that address street vending and the informal economy (Williams and Gurtoo, 2012; Ladan and Williams, 2019). Lastly, the current study is limited to one period. Future studies should, therefore, test the model using data collected at different intervals.

## Data Availability Statement

Publicly available datasets were analyzed in this study. This data can be found here: https://doi.org/10.17632/dh3cv5p7rv.1.

## Author Contributions

All authors listed have made a substantial, direct, and intellectual contribution to the work and approved it for publication.

## Funding

This project was funded by the Deanship of Scientific Research (DSR), King Abdulaziz University, Jeddah, under grant No. (DF-689-120-1441). The authors, therefore, gratefully acknowledge DSR technical and financial support.

## Conflict of Interest

The authors declare that the research was conducted in the absence of any commercial or financial relationships that could be construed as a potential conflict of interest.

## Publisher's Note

All claims expressed in this article are solely those of the authors and do not necessarily represent those of their affiliated organizations, or those of the publisher, the editors and the reviewers. Any product that may be evaluated in this article, or claim that may be made by its manufacturer, is not guaranteed or endorsed by the publisher.
